# *In Vitro* and *In Vivo* antifungal activities of selected Cameroonian dietary spices

**DOI:** 10.1186/1472-6882-14-58

**Published:** 2014-02-17

**Authors:** Jean Paul Dzoyem, Roland T Tchuenguem, Jules R Kuiate, Gerald N Teke, Frederick A Kechia, Victor Kuete

**Affiliations:** 1 Department of Biochemistry, Faculty of Science, University of Dschang, P.O. Box 67, Dschang, Cameroon; 2 Department of Biomedical Sciences, Faculty of Health Sciences, University of Bamenda, P.O. Box 39, Bambili, Cameroon

**Keywords:** Antifungal, Spices, Yeasts, Disseminated candidosis

## Abstract

**Background:**

Spices and herbs have been used in food since ancient times to give taste and flavor and also as food preservatives and disease remedies. In Cameroon, the use of spices and other aromatic plants as food flavoring is an integral part of dietary behavior, but relatively little is known about their antifungal potential.

The present work was designed to assess the antifungal properties of extracts from spices used in Cameroonian dietary.

**Methods:**

The *in vitro* antifungal activities of twenty three extracts from twenty one spices were assessed by the broth micro-dilution method against eight fungi. Also, the *in vivo* activity of *Olax subscorpioidea* extract (the most active extract) was evaluated in rat model of disseminated candidiasis due to *Candida albicans* by estimating the fungal burden in blood and kidney.

**Results:**

Seven extracts (30%) exhibited moderate to significant antifungal activities, inhibiting the growth of the microorganisms at concentrations ranging from 0.048 to 0.39 mg/mL. *Olax subscorpioidea* extract exhibited the highest antifungal activity particularly against *Candida albicans* and *Candida tropicalis* (MIC of 0.097 mg/mL and 0.048 mg/mL respectively). Sixteen extracts (70%) were weakly active (MICs > 6.25 mg/mL). Oral administration of *O. subscorpioidea* extract at the dose 2 g/kg of body weight (bw) to artificially infected rats revealed a drop in the number of colony forming units per milliliter (cfu/mL) of *Candida albicans* cells in the blood below the detection limit (100 cfu/mL) while a modest decrease was observed in the kidney.

**Conclusion:**

The present work shows that some of the spices studied possess interesting antifungal properties and could be used to treat candidiasis. Among the plant species tested, *Olax subscorpioidea* displayed the most promising result.

## Background

Nowadays, fungal diseases have emerged and are being increasingly recognized as important public health problems owing to an ever-expanding population of immuno-compromised patients [1]. Fungal infections are usually associated with *Candida*, *Aspergillus* and *Cryptococcus* species but those due to *Candida* species represent the main opportunistic fungal infections worldwide, leading to high morbidity and mortality in the population [2]. These changes are linked to the growing population of immuno-compromised patients. During the last three decades, *Candida albicans* has been the most prevalent pathogen in systemic fungal infections [3]. Presently non albicans species of *Candida* account for more than 50% of fungal infections. The opportunistic yeast mostly reported are *C. albicans, tropicalis, C. krusei, parapsilosis, C. kefyr, glabrata, C. dubliensis and C. rugosa* and *Cryptococcus neoformans* [4]. Although the antifungal active principles are diverse and numerous, only few classes of antifungal agents are currently available to treat yeast infections due to the high toxicity of many of them [5]. The high morbidity and mortality rates associated with opportunistic yeast infections indicate that current antifungal therapy to combat candidiasis is still ineffective. Furthermore, Pierce and Lopez-Ribot (2013) reported that, the current arsenal of antifungal drugs is exceedingly short and no new antifungal drugs are expected to reach the market any time soon [6]. Therefore, the discovery of new antimicrobial agents is still relevant. Among the potential sources of new agents, plants have long been investigated because they contain many bioactive compounds that can be of interest in therapy. Because of their low toxicity, there is growing interest in using spices as a source of bioactive phytochemicals for their antimicrobial properties in preventing pathogenic diseases, in addition to their flavor and fragrance qualities [7]. Many studies have confirmed the role of spices in health maintenance and promotion; the major challenge is to provide scientific evidence. Therefore intense research have been carried out over the past decade, and scientific evidence is accumulating that spices do have medicinal properties that alleviate symptoms or prevent disease [8,9]. Many studies have reported the *in vitro* activities of herbs and spices against several pathogen microorganisms. However, it has been shown that many exciting products found to be highly active on the level of the fungal cell (*in vitro* activity) were either excessively toxic or inactive when tested *in vivo*[10]. Therefore, it is essential that the potential effectiveness of the new therapy agent is also evaluated in animal models In Cameroon, the use of spices and other aromatic plants as food flavoring is an integral part of dietary behavior. To the best of our knowledge no study has investigated the effects of Cameroonian spices against eight yeast pathogens. Therefore, the present work was designed to assess the *in vitro* and *in vivo* antifungal properties of extracts from selected spices ethno-medicinally used in Cameroon.

## Methods

### Ethics statement

Animal experiments were performed in this study according to the guidelines set for the care and use of laboratory animals and with the rules formulated under the Animal Welfare Act by the United States Department of Agriculture (USDA) and by adopting ARRIVE guidelines [11].

### Plant materials and extraction

The 21 spices investigated in this work were purchased from Dschang local market, West Region of Cameroon in March 2012 (Table 1). They were identified at the Cameroonian National Herbarium where voucher specimens were deposited under the reference number quoted in Table 1. The powdered air-dried sample from each plant was separately extracted with a mixture of methanol-dichloromethane (3:1 V/V) for 48 h at room temperature. The extract was then concentrated under reduced pressure to give the crude extract. All extracts were kept at 4°C until further use.

**Table 1:** General information on the studied spices

**Name of plant (Family)**	**Ethno-medicinal uses**	**Parts used**	**Parts used in this work**	**Extraction yield (%)**	**Voucher number**
*Aframomum citratum* (Pereira) K. Schum. (ZINGIBERACEAE)	Fever, intercostals pains, tonic, aphrodisiac [12,13].	Fruits, barks and leaves	Leaves, fruits	6.12	37736/NHC
*Aframomum melegueta* (Roscoe) K. Schum. (ZINGIBERACEAE)	Constipation, fever, carminative [14].	Seeds and fruits	Fruits	3.07	39065/HNC
*Beilschmiedia cinnamomea* (Stapf) Robyns & R. Wilczek(LAURACEAE)	Not reported	Not reported	Barks	5.03	6933/SRF-Cam
*Cinnamomum zeylanicum* (Linn) Cor. (LAURACEAE)	Stimulant, antiflatulent, antiemetic; antidiarrhoeal [13].	Barks and leaves	Leaves	5.81	22309/SRF-Cam
*Dichrostachys glomerata* (Forssk.) Chiov. (MIMOSACEAE)	Headache, toothache, dysentery, elephantiasis, leprosy, syphilis, coughs and, anthelmintic, purgative, strong diuretic, epilepsy, diuretic, laxative, and the massage of fractures [15,16].	Fruits	Fruits	6.68	15220/SRF-Cam
*Dorstenia Psilirus* Welwitch (MORACEAE)	Arthralgia, cardiovascular disorders, rheumatism, snakebites, headache, stomach disorders, diuretic, tonic, stimulant, analgesic, spice [13].	Leaves and roots	Barks	16.52	44839/HNC
*Echinops giganteus* var. *lelyi* (C. D.Adams) A. Rich. (COMPOSITAE)	Heart and gastric troubles spice. Calm stomach ache, give carminative help and reduce the effects of alcohol, reduces asthma attacks [17,18].	Roots	Roots	2.76	23647/SRF-Cam
*Fagara leprieurii* (Guill and Perr) Engl (RUTACEAE)	Abdominal pain, asthma, appendicitis, toothache spice. Gastritis, gingivitis. Bilharzias, antidiarrhoeal, cancer, laxative, antimicrobial, ulcer, gonorrhea, kidney ache, sterility [19].	Barks, seeds and fruits	Fruits	5.26	37632/HNC
*Fagara macrophylla* Engl. (RUTACEAE)	Colds and stomach-ache, fever. Malaria toothache, rheumatism, and urogenital affections [13,20].	Barks, seeds and fruits	Fruits	17.63	2716/SRFK-Cam
*Imperata cylindrica* Beauv. var. *koenigii* Durand et Schinz (POACEAE)	Diuretic and anti-inflammatory agents [21].	Roots	Roots	13.07	30139/SRFK
*Mondia whitei* Hook. f. (OLACACEAE)	Aphrodisiacs, urinary tract infection, jaundice and headaches diarrhea [22].	Wholes plant, root	Bark of the roots	11.65	42920/HNC
*Monodora myristica* Dunal (ANNONACEAE)	Constipation, uterine hemorrhage, diuretic, fever [23,24].	Fruits	Fruits	9.82	2949/SFR-Cam
*Olax subscorpioidea* var. *subscorpioidea* Oliv. (OLACACEAE)	Constipation, yellow fever, jaundice, venereal diseases, Guinea worm [25].	Fruits, roots and seeds	Fruits	14.95	3528/SRFK
*Pentadiplandra brazzeana* Baill. (CAPPARACEAE)	Aphrodisiac, toothache, peptic ulcer, stomach painand hemorrhoids, diarrhea spice [25].	Barks, roots	Barks	3.81	42918/HNC
*Piper capense* L.f. (PIPERACEAE)	Sleep inducing remedy, anthelmintic, spice [13].	Fruits	Fruits	1.81	6018/SRF-Cam
*Piper guineense* Schum et Thom (PIPERACEAE)	Respiratory infections, female infertility, aphrodisiac [22].	Fruits	Fruits	10.6	6018/SRF-Cam
*Scorodophloeus zenkeri* Harms (CAESALPINIACEAE)	Headache, cough, rheumatism; constipation [19].	Barks, seeds, woods, fruits	Bark and fruits	7.67	44803/HNC
*Solanum melongena* Hierm (SOLANACEAE)	Treatment of syphilis; skin infection; gonorrhea; rheumatic disease and swollen. [14]	Fruits	Fruits	5.26	22615/SRF-Cam
*Tetrapleura tetraptera* (Schum and Thonn) Taub. (MIMOSACEAE)	Tonic, purgative, emetic, convulsions, leprosy, anti-inflammatroy, rheumatic pains, spice [13,26].	Fruits and barks	Fruits	3.71	12117/SRF-Cam
*Xylopia aethiopica* (A. Rich) Dunal (ANNONACEAE)	Headache, cough, rheumatism; constipation [19].	Barks, leaves, roots, fruits	Fruits	18.19	16419/SRF-Cam
*Xylopia parviflora* (A. Rich.) Benth (ANNONACEAE)	Stomach disorders, barrenness, headache relief, analgesic and antispasmodic [27].	Fruits	Fruits	1.45	6431/SRF-Cam

### Fungal strains and culture media

The microorganisms used in this study were *Candida albicans* ATCC 9002, *C. parapsilosis* ATCC 22019, *C. tropicalis* ATCC 750, C. *krusei* ATCC 6258, *C. lusitaniae* ATCC 200950, (American Type Culture Collection), *Cryptococcus neoformans* IP95026 (Institut Pasteur France) and two clinical isolates namely *C. guilliermondii* and *C. glabrata* (Centre Pasteur Yaounde-Cameroon)*.* Sabouraud dextrose agar (SDA) (Liofilchem Laboratories) was used for the maintenance and culture of fungal strains while Sabouraud dextrose broth (SDB) was used for the determination of the minimum inhibitory concentrations (MICs) and the minimum fungicidal concentrations (MFCs).

### Antifungal assay

#### MIC and MFC determination

The MICs were determined using the rapid 2-(4-iodophenyl)-3-(4-nitrophenyl)-5-phenyltetrazolium chloride (INT) colorimetric assay [28]. Briefly, extracts were first emulsified in DMSO/SDB (50:50 V/V). The solution obtained was then added to SDB, and serially diluted twofold making each well to have a volume of 100 μL (in a 96- wells microplate). One hundred microlitres (100 μL) of inoculum (2.4 × 10^4^ cfu/mL) prepared in SDB was then added. Wells containing SDB, 100 μL of inoculum and DMSO at a final concentration of 2.5% served as negative control. Nystatin was used as reference antifungal drugs. The plate was covered with a sterile plate sealer, then agitated to mix the contents of the wells using a shaker and incubated at 37°C for 24 h. The final concentration of DMSO was 2.5% and did not affect the microbial growth. The MICs of samples were detected after 24 h (48 h for *C. neoformans*) incubation at 37°C, following addition of 40 μL of a 0.2 mg/mL INT solution and further incubation at 37°C for 30 minutes. Viable yeast microorganisms reduce the yellow INT dye to pink. MIC was defined as the lowest sample concentration that exhibited complete inhibition of microbial growth and hence prevented this color change. The MFC was determined by adding 50 μL of the suspensions from the wells, which did not show any growth after incubation during MIC assays, to 150 μL of fresh broth. These suspensions were re-incubated at 37°C for 48 h (72 h for *C. neoformans*). The MFC was determined as the lowest concentration of extract which completely inhibited the growth of yeast.

#### Effect of *O. subscorpioidea* extract on disseminated candidiasis in rats

The *in vivo* antifungal assay was performed as described by Richardson et al., [12] and Kretschmar et al., [13] with slight modifications [29,30].

***Experimental animals:*** Pathogen-free female *albino Wistar* rats (6–8 weeks old) were used. These animals were bred in the animal house of the Department of Biochemistry, Faculty of Science of the University of Dschang.

***Induction of systemic infection with Candida albicans in rat:*** Disseminated candidiasis infection was induced by intravenous (i.v.) injection of 0.2 mL of a 10^6^ UFC/mL inoculum, prepared in sterile saline from a fresh 48 h *Candida albicans* culture to rats. Twenty-four hours after infection three animals were sacrificed to check the effectiveness of the infection by assessing the fungal load in the blood and kidneys. This in vivo study conformed to animal research: reporting in vivo experiment guideline for reporting animal study (ARRIVE).

***Antifungal treatment***: Infected rats distributed into five groups of 12 animals each were housed in cages and had access to food and water *ad libitum*. Extract at the doses of 0.5, 1 and 2 g/kg of bw, were administered i.v. over three consecutive days, starting 24 h after infection. Two control groups were used; untreated control received distilled water and a positive control group treated with reference antifungal drug nystatin at 10 mg/kg of bw.

***Determination of viable yeasts in organ samples:*** Twenty-four hours after the first, second and third treatment (at days 2, 3 and 4 of infection) three animals in each group were sacrificed by cervical dislocation; blood and organ samples were collected from each rat. Kidney tissues were homogenized in 5 mL of sterile saline, and then serially diluted. Blood samples were also serially diluted. 0.1 mL of each dilution was plated onto SDA containing chloramphenicol, incubated for 24 h at 37°C and the number of fungal colony were counted. The lower limit of detection was 100 cfu/mL. The results were expressed as log of the mean number of cfu/g of tissue per three animals.

### Statistical analysis

One way analysis of variance was used to analyze the fungal load in animal tissues between each treatment group and the control group. When there were differences between groups, the means were compared using the Student-Newman-Keuls test at a 5%. Results are expressed as mean ± standard deviation. All data were analyzed using SPSS Statistics 17.0.2.

## Results and discussion

***In vitro *** **antifungal activity:** Spices have been used for generations by humans as food and to treat ailments. As depicted in Table 1, spices used in this study are used traditionally in the treatment of a wide range of diseases. Many studies have confirmed their role in health maintenance and promotion, but the major challenge is to provide scientific evidence [7]. Therefore, the *in vitro* and *in vivo* antifungal potentials of 23 extracts from spices used in Cameroonian dietary were investigated. The results of *in vitro* antifungal assay (MIC and MBC) are presented in Table 2. The results indicated that all the extracts demonstrated both fungistatic and fungicidal activities with the minimum inhibitory concentration ranging from 0.048 to 6.25 mg/mL and the minimum fungicidal concentration ranging from 0.19 to 6.25 mg/mL. Each of the extract tested in the present study displayed antifungal activity on at least one of the 8 pathogen yeasts tested. However differences were observed between antifungal activities as most of the tested plant extracts exerted a broad antifungal spectrum. These variations in antifungal activity could be due to the differences in the chemical composition of these spices as the secondary metabolites of plants have many effects including antimicrobial properties [31]. In addition, the activity of the plant extract could be influenced by the nature of the plant material or its origin as well as the climatic conditions in which plant grow, the plant part used, or the solvent used for extraction, because plants have different constituents depending on those factors [32].

**Table 2:** MIC and MFC (mg/mL) of 23 extracts from 21 Cameroonian spices against eight opportunistic yeasts fungi

**Tested samples**	**Microorganisms**															
	***Ca***		***Cn***		***Ct***		***Ck***		***Cp***		***Cl.***		***Cgu.***		***Cgl***	
	**MIC**	**MFC**	**MIC**	**MFC**	**MIC**	**MFC**	**MIC**	**MFC**	**MIC**	**MFC**	**MIC**	**MFC**	**MIC**	**MFC**	**MIC**	**MFC**
*A. citratum* (fr)	-	-	6.25	-	6.25	-	-	-	6.25	-	0.78	1.56	3.12	6.25	6.25	-
*A. citratum* (le)	3.12	-	**0.19**	6.25	6.25	6.25	**0.39**	6.25	6.25	-	0.78	0.78	3.12	-	3.12	-
*A. melegueta*	6.25	-	6.25	-	1.56	1.56	6.25	-	6.25	-	3.12	3.12	1.56	-	-	-
*B. cinnamomea*	3.12	3.12	3.12	6.25	3.12	-	-	-	-	-	3.12	-	6.25	-	6.25	6.25
*C. zeylanicum*	3.12	-	1.56	3.12	**0.097**	**0.19**	0.78	6.25	3.12	6.25	0.78	0.78	0.78	0.78	3.12	-
*D. glomerata*	**0.39**	6.25	0.78	-	3.12	3.12	3.12	3.12	6.25	-	**0.39**	6.25	3.12	6.25	3.12	6.25
*D. psilirus*	**0.39**	1.56	6.25	-	**0.39**	1.56	3.12	3.12	3.12	3.12	3.12	3.12	1.56	1.56	3.12	-
*E. giganteus*	6.25	-	3.12	-	6.25	6.25	-	-	6.25	6.25	6.25	6.25	3.12	-	1.56	-
*F. leprieurii*	3.12	6.25	6.25	6.25	6.25	-	6.25	-	6.25	-	-	-	-	-	3.12	-
*F. macrophylla*	3.12	6.25	1.56	3.12	**0.39**	0.78	6.25	-	-	-	1.56	6.25	1.56	-	3.12	-
*I. cylindrica*	6.25	-	1.56	1.56	3.12	-	1.56	1.56	6.25	-	3.12	3.12	3.12	-	3.12	6.25
*M. whitei*	3.12	-	-	-	3.12	-	-	-	-	-	3.12	6.25	6.25	-	-	-
*M. myristica*	6.25	-	0.78	6.25	3.12	3.12	6.25	6.25	-	-	3.12	6.25	3.12	-	-	-
*O. subscorpioidea*	**0.097**	**0.19**	**0.39**	1.56	**0.048**	**0.19**	1.56	6.25	**0.39**	1.56	**0.19**	**0.39**	0.78	-	1.56	6.25
*P. brazzeana*	3.12	6.25	6.25	6.25	1.56	1.56	-	-	-	-	6.25	-	6.25	-	6.25	-
*P. capense*	3.12	-	1.56	1.56	**0.19**	3.12	3.12	6.25	3.12	6.25	1.56	1.56	3.12	-	-	-
*P. guineense*	3.12	3.12	3.12	-	3.12	-	-	-	6.25	-	1.56	-	-	-	3.12	-
*S. zenkeri* (fr)	1.56	-	-	-	6.25	-	6.25	-	6.25	6.25	1.56	6.25	6.25	-	3.12	6.25
*S. zenkeri* (bk)	3.12	-	3.12	-	3.12	-	1.56	6.25	6.25	-	3.12	6.25	6.25	-	6.25	-
*S. melongena*	-	-	3.1-	-	3.12	3.12	-	-	-	-	-	-	6.25	6.25	-	-
*T. tetraptera*	3.12	-	3.12	-	6.25	6.25	6.25	-	3.12	6.25	3.12	-	3.12	-	6.25	-
*X. aethiopica*	3.12	6.25	6.25	6.25	3.12	6.25	3.12	3.12	3.12	3.12	-	-	-	-	-	-
*X. parviflora*	6.25	-	-	-	3.12	-	3.12	3.12	3.12	3.12	1.56	1.56	3.12	6.25	6.25	-
*Nystatin* (μg/mL )	78.1	31.2	195.3	78.1	4.8	19.5	6-	1-0	39	156	19.5	78.1	39	78.1	19.5	39

*Ca: Candida albicans*, *Cn: Cryptococcus neoformans, Ct: Candida tropicalis*, *Ck: Candida krusei, Cp: Candida parapsilosis*, *Cl: Candida lusitaniae*, *Cgu: Candida guilliermondii, Cgl: Candida glabrata,* fr: fruits, le: leaves, bk: barks. -: > 6.25 mg/mL, le: leaves, fr: fruits, bk: barks, In bold are values for significant activity.

Antimicrobial activity of plant extracts are routinely classified on the basis of susceptibility tests that produce MIC in the range of 100 to 1000 μg/mL [33]. The activity is considered to be significant if MIC values are below 100 μg/mL for crude extract and moderate when the MICs vary from 100 to 625 μg/mL [15,32]. Seven extracts (30%) inhibited the growth of 6 studied yeasts from significant to moderated extent while other extracts exhibited weak activities. These results are supported by previous studies reporting that the commonly used herbs and spices possess antimicrobial properties that, in some cases, can be used therapeutically [8]. Considering the fact that the spices tested are used as food ingredients with limited toxicity, the overall activity recorded with several extracts, especially those of *A. citratum* (leaves)*, D. glomerata, D. psilirus, F. macrophylla and P. capense*, could be considered important. The best activity was observed with the extract from *O. subscorpioidea* fruits showing significant to moderate activity against five out of the eight opportunistic yeast strains tested, with a particular strong effectiveness against *Candida albicans* and *Candida tropicalis* (MIC of 0.097 mg/mL and 0.048 mg/mL respectively). The extracts from *C. zeylanicum* leaves also showed significant activity against *Candida tropicalis* (MIC of 0.097 mg/mL). High antimicrobial activities of *C. zeylanicum* against clinical strains of *C. albicans* from nosocomial infections were previously reported [34]. Qualitative phytochemical analysis of *O. subscorpioidea* and *C. zeylanicum* revealed the presence of alkaloids, anthraquinones, phenols, flavonoids, tannins and triterpernes which could contribute to the observed antifungal activities [35]. Many of the phytonutrients found in spices act as potent preventive agents against microbial diseases. It is generally found that plants containing diverse classes of chemicals are of superior biological activities [11]. Thus, it may also be deduced that the higher the diversity and the quantity of these chemical classes a plant may contain, the stronger and broader the spectrum of biological activities the plant molecules may exhibit [36].

Among the eight pathogenic yeasts tested *C. albicans* and *Candida tropicalis* were the most sensitive to the extracts, while *C. krusei* and *C. glabrata* were the most resistant. Since *C. albicans* is the most prevalent pathogen in systemic fungal infections [3] and being the most susceptible to *O. subscorpioidea* extract (MIC of 0.097 mg/mL) *in vitro*, the *in vivo* therapeutic effect of *O. subscorpioidea* extract was evaluated on systemic infection induced with *C. albicans* in rats.

**Figure 1:**
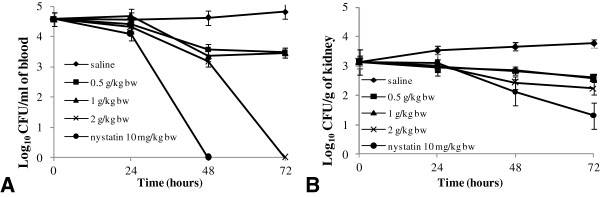
**Variation of fungal burden of blood (A) and kidney (B) in rat model of disseminated candidiasis at various time intervals upon treatment with extract *Olax subscorpioidea *and nystatin.** Animals were infected intravenously with 10^6^ cells/mL(Candida *albicans*) and were treated for three consecutive days with saline, extract (0.5, 1 and 2 g/kg of bw) and nystatin (10 mg/kg of bw). The fungal burden was assessed by determining the number of cfu.

***In vivo *** **antifungal activity:** The intravenous challenge model was used to study invasive *Candida* infection. Yeast cells were injected directly into the blood stream through the lateral tail vein [37]. In this model, which is similar to human invasive infection occurring with catheter involvement, fungal cells are found in all organs, but disease progresses mostly in the kidneys and brain. It is well known that the kidney is the primary target organ in systemic candidiasis; it is the major organ in which the multiplication of *Candida* occurs [38]. Therefore, the *in vivo* activity of extract from *O. subscorpioidea* was evaluated in experimental systemic candidiasis with *C. albicans* in rats, by estimating the fungal burden in this organ as well as in blood. The results on the estimated number of viable *C. albicans* cells in blood and kidneys from animals that were infected intravenously with 1×10^6^ cells/mL and treated for three consecutive days with saline, extract (0.5, 1 and 2 g/kg of bw) are shown in Figures 1. The administration of the extract caused a reduction of the fungal load after 24 h both in the blood and in kidneys of infected rats compare to untreated control. A significant difference was observed (*p* < 0.05) in the decrease of fungal load in blood at 2 g/mg of bw with respect to the control (Figure 1A). At this extract concentration, the number of cfu/mL in blood dropped below the detection limit within 72 h, while the control (nystatin) showed similar effect within 48 h. This result suggests that *C. albicans* cells intravenously injected were rapidly cleared from the blood. The rapid disappearance of *C. albican*s from the blood observed could be attributed to the combined killing effect of extract or nystatin and the immune system cells. Our findings are in agreement with those of Robert et al. [39], who reported that after injection into the lateral tail veins of mice, the rate of clearance of *C. albicans* from the bloodstream was high, leading to the disappearance of 97% of the inoculated cells within the first minute. It should also be noted that this observation could be explained further by the fact that some of the *C. albicans* cells moved into the deep organs like the kidneys, being the primary target organ in systemic candidiasis where the multiplication of *C. albicans* occurs [38]. As shown in Figure 1B, compared to untreated infected animals, extract reduced the level of fungal load in kidneys during treatment. After 3 days of treatment with extract each of the test doses modestly reduced the growth of fungi in the kidney. At the highest dose (2 g/kg of bw), reduction in the log of cfu/g in the kidney was more pronounced compared to the lower doses. Although the reduction was not statistically significant, the result obtained is important when compared to those obtained with nystatin. Nevertheless, it is noteworthy that the result obtained could be considered as significant bearing in mind that *C. albicans* survival in the kidney plays a primary role in mortality in patients with disseminated candidiasis [40]. Our results revealed that extract from *O. subscorpioidea* displayed an inhibitory effect on fungal proliferation *in vivo*. To the best of our knowledge, the *in vivo* antifungal properties of spices are herein reported for the first time. Therefore, a subject for future investigation with a possible prolongation of the period of treatment to 7 or 14 days to more accurately define the preclinical significance of the *in vivo* data herein observed.

The overall results obtained revealed that, extract from spices in this study possess inhibitory effect on the growth of pathogenic yeast. *Olax subscorpioidea* showed the most promising activity *in vitro* and exerted interesting anti-Candida effect against systemic candidiasis in rat model. These findings emphasize the evidence that increasing our intake in spices could be an alternative to prevention of infections from opportunistic fungal yeast pathogens.

## Conclusion

The present study demonstrates the antifungal potential of extracts from some Cameroonian spices and provides the scientific evidence that natural spices could be used for the prevention of yeast diseases.

## Competing interests

The authors declared that they have no competing interests.

## Authors’ contributions

JPD designed the experiments and wrote the manuscript; RTT and GNT performed the experiments. JPD, FAK, VK and JRK supervised the work. All authors read and approved the final manuscript.

## Pre-publication history

The pre-publication history for this paper can be accessed here:

http://www.biomedcentral.com/1472-6882/14/58/prepub

## References

[B1] Miceli MH Diaz JA Lee SA Emerging opportunistic yeast infections Lancet Infect Dis 2011 11 142 151 10.1016/S1473-3099(10)70218-8 21272794

[B2] Low CY Rotstein C Emerging fungal infections in immunocompromised patients F1000 Med Rep 2011 3 14 21876720 10.3410/M3-14 PMC3155160

[B3] Pfaller MA Diekema DJ Rare and emerging opportunistic fungal pathogens: concern for resistance beyond *Candida albicans* and *Aspergillus fumigatus* J Clin Microbiol 2004 42 4419 4431 10.1128/JCM.42.10.4419-4431.2004 15472288 PMC522363

[B4] Banerje U Satyanarayana T, Kunze G Opportunistic pathogenic yeasts Yeast Biotechnology: Diversity and Applications. Part I 2009 Netherlands: Springer 215 236

[B5] Spampinato C Leonardi D *Candida* infections, causes, targets, and resistance mechanisms: traditional and alternative antifungal agents BioMed Res Int 2013 204237 23878798 10.1155/2013/204237 PMC3708393

[B6] Pierce CG Lopez-Ribot JL Candidiasis drug discovery and development: new approaches targeting virulence for discovering and identifying new drugs Expert Opin Drug Discov 2013 8 1117 1126 10.1517/17460441.2013.807245 23738751 PMC3984500

[B7] Arora DS Kaur J Antimicrobial activity of spices Int J Antimicrob Agents 1999 12 257 262 10.1016/S0924-8579(99)00074-6 10461845

[B8] Lai PK Roy J Antimicrobial and chemopreventive properties of herbs and spices Curr Med Chem 2004 11 1451 1460 10.2174/0929867043365107 15180577

[B9] Naser AAW Antimicrobial and antioxidant properties of spices Bull Pharm Sci, Assiut University 2007 30 81 87

[B10] Polak A Experimental models in antifungal chemotherapy Mycoses 1998 41 1 30 9610129 10.1111/j.1439-0507.1998.tb00371.x

[B11] Kilkenny C Browne W Cuthill IC Emerson M Altman DG Animal research: reporting in vivo experiments_the ARRIVE guidelines J Cereb Blood Flow Metab 2011 31 991 993 10.1038/jcbfm.2010.220 21206507 PMC3070981

[B12] Tane P Tatsimo SD Ayimele GA Connolly JD Bioactive metabolites from Aframomum species 2005 Antananarivo, Madagascar: 11th NAPRECA Symposium Book of Proceedings 214 223

[B13] Kuete V Krusche B Youns M Voukeng I Fankam AG Tankeo S Lacmata S Efferth T Cytotoxicity of some Cameroonian spices and selected medicinal plant extracts J Ethnopharmacol 2011 134 803 812 10.1016/j.jep.2011.01.035 21291988

[B14] Dalziel JM The useful plants of west tropical Africa 1937 London: Crown Agents for the Colonies

[B15] Kuete V Potential of Cameroonian plants and derived products against microbial infections: a review Planta Med 2010 76 1479 1491 10.1055/s-0030-1250027 20533165

[B16] Gisèle GAK Dzeufiet DD Foyet HS Nana P Sokeng CD Dimo T Kamtchouing P Analgesic and anti-inflammatory activities of *Dichrostachys glomerata* (forssk.) hutch. fruits methanolic extract in rats J Phys Pharm Adv 2012 2 269 276

[B17] Menut C Lamaty G Weyerstahl P Marschall H Seelmann I Amvam Zollo PH Aromatic plants of tropical central Africa. Part XXXI. Tricyclic sesquiterpenes from the root essential oil of *Echinops giganteus* var. *lelyi* C. D. Adams Flavour Fragrance J 1997 12 415 421 10.1002/(SICI)1099-1026(199711/12)12:6<415::AID-FFJ666>3.0.CO;2-T

[B18] Tene M Tane P Sondengam BL Connolly JD Lignans from the roots of *Echinops giganteus* Phytochemistry 2004 65 2101 2105 10.1016/j.phytochem.2004.05.014 15279979

[B19] Ngono Ngane A Biyiti L Amvam Zollo PH Bouchet P Evaluation of antifungal activity of extracts of two Cameroonian Rutaceae: *Zanthoxylum leprieurii* Guill. et Perr. and *Zanthoxylum xanthoxyloides* Waterm J Ethnopharmacol 2000 70 335 342 10.1016/S0378-8741(99)00188-9 10837996

[B20] Irvine FR Woody plants of Ghana 1961 Oxford University Press

[B21] Nishimot K Ito M Natori S Ohmoto T Structures of Arundoin Cylindrin and Fernenol - Triterpenoids of Fernane and Arborane Groups of *Imperata Cylindrica* Var *Koenigii* Tetrahedron 1968 24 735 752 10.1016/0040-4020(68)88023-8

[B22] Noumi E Zollo PHA Lontsi D Aphrodisiac plants used in Cameroon Fitoterapia 1998 69 125 134

[B23] Tatsadjieu LN Ngang JJE Ngassoum MB Etoa FX Antibacterial and antifungal activity of *Xylopia aethiopica*, *Monodora myristica*, *Zanthoxylum xanthoxyloides* and *Zanthoxylum leprieurii* from Cameroon Fitoterapia 2003 74 469 472 10.1016/S0367-326X(03)00067-4 12837363

[B24] Okafor JC Development of forest tree crops for food supplies in Nigeria Forest Ecol Manag 1978 1 235 247

[B25] Okoli RI Aigbe O Ohaju-Obodo JO Mensah JK Medicinal herbs used for managing some common ailments among Esan people of Edo State, Nigeria Pak J Nutr 2007 6 490 496 10.3923/pjn.2007.490.496

[B26] Tekwu EM Askun T Kuete V Nkengfack AE Nyasse B Etoa F Beng VP Antibacterial activity of selected Cameroonian dietary spices ethno-medically used against strains of *Mycobacterium tuberculosis* J Ethnopharmacol 2012 142 374 382 10.1016/j.jep.2012.05.003 22595661

[B27] Nishiyama Y Moriyasu M Ichimaru M Iwasa K Kato A Mathenge SG Mutiso PBC Juma FD Antinociceptive effects of the extracts of *Xylopia parviflora* bark and its alkaloidal components in experimental animals J Nat Med-Tokyo 2010 64 9 15 10.1007/s11418-009-0356-2 19730974

[B28] Eloff JN A sensitive and quick microplate method to determine the minimal inhibitory concentration of plant extracts for bacteria Planta Med 1998 64 711 713 10.1055/s-2006-957563 9933989

[B29] Richardson K Brammer KW Marriott MS Troke PF Activity of Uk-49,858, a Bis-triazole derivative, against experimental infections with *Candida albicans* and *Trichophyton mentagrophytes* Antimicrob Agents Chemother 1985 27 832 835 10.1128/AAC.27.5.832 2990328 PMC180161

[B30] Kretschmar M Amselem S Zawoznik E Mosbach K Dietz A Hof H Nichterlein T Efficient treatment of murine systemic infection with *Candida albicans* using amphotericin B incorporated in nanosize range particles (emulsomes) Mycoses 2001 44 281 286 10.1111/j.1439-0507.2001.00654.x 11714063

[B31] Cowan MM Plant products as antimicrobial agents Clin Microbiol Rev 1999 12 564 582 10515903 10.1128/cmr.12.4.564 PMC88925

[B32] Ncube NS Afolayan AJ Okoh AI Assessment techniques of antimicrobial properties of natural compounds of plant origin: current methods and future trends Afr J Biotechnol 2008 7 1797 1806

[B33] Simoes M Bennett RN Rosa EAS Understanding antimicrobial activities of phytochemicals against multidrug resistant bacteria and biofilms Nat Prod Rep 2009 26 746 757 10.1039/b821648g 19471683

[B34] Khan R Islam B Akram M Shakil S Ahmad A Ali SM Siddiqui M Khan AU Antimicrobial activity of five herbal extracts against multi drug resistant (MDR) strains of bacteria and fungus of clinical origin Molecules 2009 14 586 597 10.3390/molecules14020586 19214149 PMC6253777

[B35] Fankam AG Kuete V Voukeng IK Kuiate JR Pages JM Antibacterial activities of selected Cameroonian spices and their synergistic effects with antibiotics against multidrug-resistant phenotypes BMC Complement Altern Med 2011 11 104 10.1186/1472-6882-11-104 22044718 PMC3228721

[B36] Wangchuk P Keller PA Pyne SG Taweechotipatr M Tonsomboon A Rattanajak R Kamchonwongpaisan S Evaluation of an ethnopharmacologically selected Bhutanese medicinal plants for their major classes of phytochemicals and biological activities J Ethnopharmacol 2011 137 730 742 10.1016/j.jep.2011.06.032 21741462

[B37] Papadimitriou JM Ashman RB The pathogenesis of acute systemic candidiasis in a susceptible inbred mouse strain J Pathol 1986 150 257 265 10.1002/path.1711500405 3806283

[B38] Baine WB Koenig MG Goodman JS Clearance of *Candida albicans* from blood-stream of rabbits Infect Immun 1974 10 1420 1425 4611929 10.1128/iai.10.6.1420-1425.1974 PMC423120

[B39] Robert R Nail S Marot-Leblond A Cottin J Miegeville M Quenouillere S Mahaza C Senet JM Adherence of platelets to *Candida* species *in vivo* Infect Immun 2000 68 570 576 10.1128/IAI.68.2.570-576.2000 10639419 PMC97178

[B40] Bendel CM Kinneberg KM Jechorek RP Gale CA Erlandsen SL Hostetter MK Wells CL Systemic infection following intravenous inoculation of mice with *Candida albicans* int1 mutant strains Mol Genet Metab 1999 67 343 351 10.1006/mgme.1999.2875 10444345

